# Femoral Stress Changes after Total Hip Arthroplasty with the Ribbed Prosthesis: A Finite Element Analysis

**DOI:** 10.1155/2020/6783936

**Published:** 2020-03-23

**Authors:** Changqi Luo, Xiang-Dong Wu, Yifei Wan, Junyi Liao, Qiang Cheng, Mian Tian, Zhibiao Bai, Wei Huang

**Affiliations:** ^1^Department of Orthopedic Surgery, The First Affiliated Hospital of Chongqing Medical University, Chongqing 400016, China; ^2^Department of Orthopaedic Surgery, Peking Union Medical College Hospital, Chinese Academy of Medical Sciences & Peking Union Medical College, Beijing 100730, China; ^3^School of Mechanical Engineering, Southwest Jiaotong University, Chengdu 610031, China; ^4^Department of Orthopaedic Surgery, Dianjiang People's Hospital, Chongqing 408300, China

## Abstract

**Background:**

A total hip reconstruction is related to the stress distribution throughout the prosthesis, cement, and femur. Researches on reducing the stress in all components to minimize the risk of failure are of great significance. The objective of our study was to determine the biomechanical variation in overall femoral stress and periprosthetic femoral stress distribution after implantation with the Ribbed anatomic prosthesis.

**Methods:**

Three-dimensional finite element models of intact femur and Ribbed prosthesis were developed according to the morphology, while the hip joint loading and the strength of related muscles were applied in the models. The overall stress changes of the intact femur before and after the implantation were analyzed, and the periprosthetic stress distribution especially in the proximal region of the femur was quantified.

**Results:**

As a result, the overall stress pattern of the femur did not change after the implantation compared with the intact femur. The region of peak stress value was located in the middle and lower segments of the full length femur, but the stress value level decreased. The final prosthesis resulted in a significant decrease in the equivalent stress level of the periprosthetic bone tissue, and the most severe area appeared at the endmost posterior quadrant. The stress shielding ratio of the Ribbed prosthesis was 71.6%. The stress value level gradually increased towards the distal part of the prosthesis and recovered to physiological level at the end of the prosthesis.

**Conclusions:**

The Ribbed prosthesis can cause significant stress shielding effect in the proximal femur. These results may help optimize prosthetic design to reduce stress shielding effect and improve clinical outcomes.

## 1. Introduction

Total hip arthroplasty (THA) is a primary treatment for advanced hip diseases such as severe hip osteoarthritis and avascular necrosis of the femoral head. Each year, over a million patients undergo this operation around the world [1–4]. Unfortunately, about 10% of patients remain not satisfied with the treatment effect [5–7]. The key problems to be solved in THA are the nonuniform stress transfer of each component and the structural compatibility between the prosthesis and the femoral canal. The force line of the artificial hip joint is transmitted from the pelvis to the proximal femur through the femoral head, the femoral neck, and the femoral stem [8–12]. Good force transmission can avoid femoral stress shielding effect, so that the femoral stem and the proximal femur can be fully fitted to make the femoral stem more stable [13]. Wolff's law described the optimal structure of bone formation to carry and adapt to load changes, and the changes in the stress distribution and conduction of local bone tissue will cause a rebalancing of osteogenesis and osteoclast activity [4, 14, 15]. However, radiography examination revealed that severe bone loss in the proximal region due to bone remodeling was considered to be one of the causes of prosthesis loosening after THA [16].

According to the basic design concepts of cementless femoral stems, the main rationales in stem geometry can be classified into three types: anatomic designs, straight designs, and tapered designs. Although anatomic stems were designed to match the shape of the femoral cavity as much as possible, the difference in anatomical features and the stress distribution in the local area after implantation directly affect the postoperative femur-stem integration, bone remodeling, and mechanical transmission, thus leading to aseptic loosening of the hip prosthesis. The Ribbed prosthesis is designed anatomically S-shaped to realize insertion of the maximum allowable stem size and to reduce the rotational forces affecting the prosthetic anchorage. The stem is also designed with deep grooves to increase the modulus of elasticity and to reduce the stress shielding effect or excessive stiffening of the proximal femur caused by the metallic implant. Moreover, an anchoring screw through a bore hole in the lateral fin can be screwed into the greater trochanter to reduce the compressive load onto the calcar during the initial postoperative stage [17]. At present, there are few studies on the features of stress distribution after THA with Ribbed prosthesis. Whether it solves the above problems well may require further confirmation. Therefore, fully understanding the mechanism is of great importance to optimize the prosthetic design and to improve clinical outcomes.

Finite element analysis (FEA) is one of the important methods regarding stress studies. It can treat countless mass points and continuums of infinite degrees of freedom as a collection of approximately finite elements. The finite elements are hinged on the nodes to form an aggregate with a finite number of degrees of freedom. The FEA was first used in the analysis of structural mechanics and later introduced into the study of orthopedic biomechanics [18, 19]. With the rapid development of computer and digital technology, it is called a multipurpose tool for biomechanical research [20]. In view of the complex structure of the hip joint, biomechanical experiments are not safe and accurate [21, 22]. In addition, the FEA enables repeated experiments to measure internal and local mechanical responses that cannot be measured in general biomechanical experiments. Stress distribution characteristics under the condition of internal fixation with fixed instrument loading can be definitely analyzed by using this method.

Therefore, the objective of our study was to determine the biomechanical variation in overall femoral stress and periprosthetic femoral stress distribution after implantation with the Ribbed anatomic prosthesis. The femoral stress distribution before and after Ribbed anatomic prosthesis implantation was quantified, and the changes in biomechanical environment were analyzed and observed.

## 2. Materials and Methods

Ethical approval for the study was obtained through the ethics committee of the First Affiliated Hospital of Chongqing Medical University (IRB 2017-187-2).

### 2.1. Radiography Data

**Figure 1:**
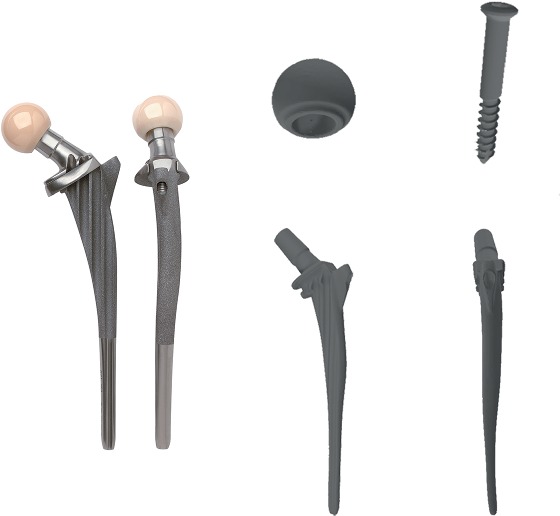
The Ribbed anatomic hip system and the matched type in STL format.

Even if there are techniques to clear off the metal artifacts, it remains difficult to accurately analyze the state of the implant by the FEA. Therefore, a contralateral femur was scanned by spiral CT with a layer thickness of 0.625 mm. The scanned imaging data was output in DICOM format and saved in a computer. Meanwhile, the prosthesis system which matches with it including modular stem, head, detachable collar, and anchoring screw in the lateral fin (Ribbed® Hip System, Waldemar Link®, Hamburg, Germany) was scanned by a three-dimensional laser scanner. The data was output in STL format as shown in Figure 1.

### 2.2. Establishment of the Three-Dimensional Finite Element Model

**Figure 2:**
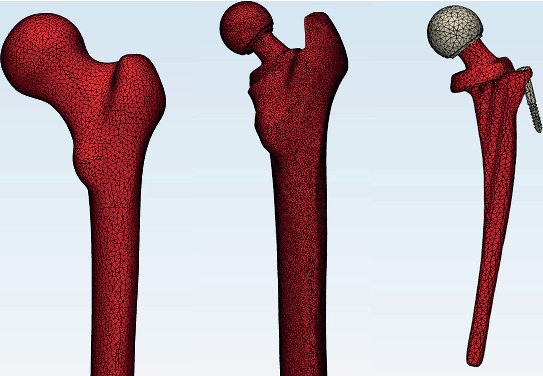
The meshing diagram of finite element models of the femur inserted with Ribbed anatomic prosthesis.

The DICOM data was imported into the software Mimics Medical version 20.0 (Materialise Inc., Belgium) to reconstruct original three-dimensional models, and all of them were assembled after surface optimization by the software Geomagic Studio version 2015 (Geomagic Corporation, NC, USA). After the prosthesis was assembled according to the standard position of the operation manual to establish a postoperative model, the cortical bone, cancellous bone, and femoral prosthesis were meshed with tetrahedral elements. Then, the finite element model was built into the software ANSYS version 19.0 (ANSYS Inc., USA). In the end, there were 40285 nodes of cancellous bone, 170401 units; 35167 nodes of cortical bone, 132580 units; 14107 nodes of prosthesis stem, 47604 units; and 1340 nodes of metal head, 4407 units (Figure 2).

### 2.3. Material Properties and Application of Load

Each part of the bone model was divided according to the physiological distribution of cortical bone and cancellous bone, and the properties of each material were based on the data proposed by Stolk et al. [23] (Table 1). The cortical bone is a transversely isotropic elastic material, while the cancellous bone is an isotropic elastic material. According to the specification, the prosthetic head and the main body are made of CoCrMO alloy and titanium, respectively. As for the joint force of the hip and muscle, one-legged standing condition was simulated. A load of 2400 N was applied to the femoral head at an angle of 16° relative to the femoral axis, and a load of 1200 N was applied to the greater trochanter at an angle of 21° [24, 25]. With regard to the prosthesis implant model, the interface state after the stable bone ingrowth was simulated, and the degrees of freedom of the prosthesis-bone interface node were coupled. During the analysis, the distal region of the femur was completely fixed, and all 6 degrees of freedom were constrained.

### 2.4. Femoral Stress Partition

**Figure 3:**
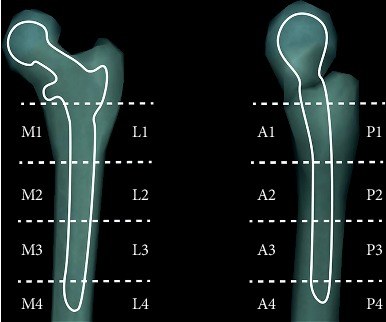
Graph of the four horizontal segments and quadrants of stress distribution in the proximal femoral regions.

For the purpose of quantifying the proximal femoral stress, the periprosthetic femur was divided into four horizontal segments, representing the proximal, middle, distal, and endmost regions. Each segment was divided into four quadrants according to the anterior, posterior, medial, and lateral directions (Figure 3). The average stress of all nodes in each quadrant was taken as the stress level of the region.

## 3. Results

### 3.1. Overall Stress Distribution of the Femur

**Figure 4:**
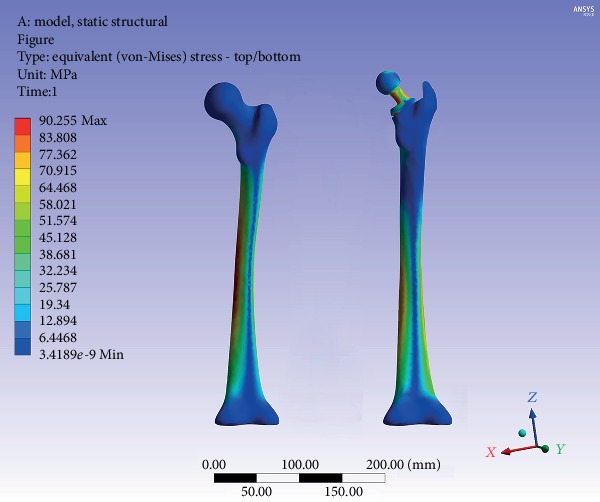
The von Mises stress nephogram of the intact femur and inserted with prosthesis.

**Figure 5:**
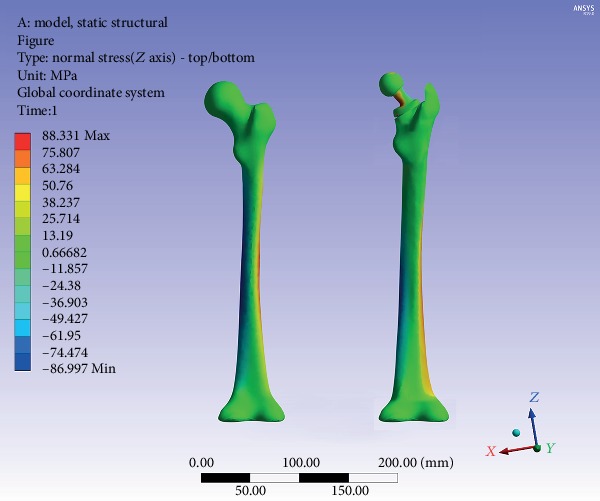
Axial stress nephogram of the intact femur and inserted with prosthesis.

The equivalent stress distribution of the femur before and after implantation of the Ribbed stem is shown in Figure 4. The intact femoral stress level gradually increased from the proximal to the distal region and reached the peak level in the middle and distal parts of the femur. The maximum value of stress was 90.3 MPa. On the other hand, the overall stress pattern of the femur was almost unchanged after implantation. The peak stress region was still in the middle and distal parts of the femur, but the maximum value decreased to 87.5 MPa. The axial stress distribution of the femur before and after implantation of the Ribbed stem is shown in Figure 5.

The compressive stress was predominant in the medial part of the intact femur, while the tensile stress mainly appeared in the lateral part. The compressive stress value was lower than the tensile stress (87.0 MPa vs. 88.3 MPa, respectively). As previously described, the axial stress pattern of the femur still did not change significantly after implantation, and the maximum value of the compressive stress and tensile stress decreased to 76.5 MPa and 73.2 MPa in the *Z*-axis direction, respectively.

### 3.2. Proximal Stress Distribution of the Femur

The change of equivalent stress distribution of the proximal femur was most significant after implantation. To quantify the variation before and after the implantation, the stress values of 16 regions in the proximal femur were calculated according to the formula to get the stress shielding ratio (*μ*) of the corresponding region after implantation (*ε* and *ε*_0_ represented postoperative and preoperative equivalent stresses, respectively).

(1) $$\begin{array}{c} \mu =\left(\frac{\varepsilon -\varepsilon_{0}}{\varepsilon_{0}}\right)\times 100\%. \end{array}$$

**Figure 6:**
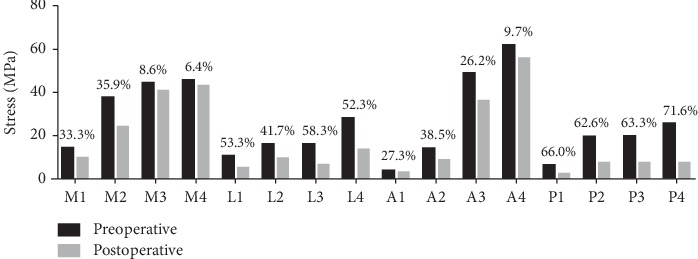
Equivalent stress distribution of the proximal femur before and after implantation and corresponding stress shielding ratio.

The stress values of each region and the stress shielding ratio are explicitly shown in Figure 6.

The stress level in the medial and anterior quadrants of the intact proximal femur was higher than that in the lateral and posterior quadrants. The stress level in the anterior, posterior, and medial quadrants gradually increased from the proximal to the distal region, while region A4 showed high stress with a value of 61.8 MPa.

The stress in the proximal femur decreased significantly after implantation of the prosthesis, and the stress level in four quadrants gradually increased from the proximal to the distal region. To be specific, obvious stress shielding effect was found in the lateral and posterior regions, and the most severe region was the endmost posterior quadrant (P4) with a stress shielding ratio of 71.6%. However, the minimum stress shielding ratio was only 6.4%, which appeared in the endmost medial quadrant (M4).

## 4. Discussion

To the best of our knowledge, the forces acting on the femoral head and its surrounding structure in hip joint activity include compressive and bending properties, and the combined action of the two forces significantly affects the stress distribution in the proximal part of the femur. It is troubling that stress shielding and stress concentration after hip arthroplasty are one of the main reasons of prosthesis loosening and sinking [26–28]. These effects are caused by the mismatch between the mechanical properties of the prosthesis and the surrounding structure. If the artificial hip joint is to achieve favorable force transmission, the matching degree is an important consideration. Proximal matching can reduce the stress shielding effect, allowing the femoral stem to transmit force to the surrounding structure without causing bone resorption, while distal matching will increase the stability of the femoral stem [29, 30]. Therefore, how to achieve an optimum design depends on all those factors to maintain stability between the stem and the bone and avoiding complications after THA as well. Plenty of studies have followed up the postoperative clinical outcomes of the Ribbed prosthesis in many clinical centers, but few literatures on the features of them from a perspective of design are reported [31–36]. Fortunately, we are the first to quantify stress distribution before and after implantation by FEA aiming to optimize the prosthetic design and improve clinical outcomes.

In the present study, the prosthesis and femur have constituted a new mechanical system. We have observed that the intact femoral stress level gradually increased from the proximal to the distal region and reached the peak level in the middle and lower parts of the femur. It should be remembered that the phenomenon may be related to the bending moment effect of the femur. Most studies suggested that bending moment produces compressive stress on the medial side of the femur and tensile stress on the lateral side. Nevertheless, the perspective contradicts the work by Taylor et al. that muscle strength and anterior arch of the femur resist the bending moment effect, and the bilateral loads of the femur are mainly compressive stress [37]. In this study, it was considered that the muscle strength was not sufficient to resist all bending moment, and there was high compressive stress on the medial side of the femur but low tensile stress on the lateral side. Obviously, the stress pattern did not change after implantation of the prosthesis, while the maximum stress value decreased significantly. That was to say, the bending moment effect after implantation was weakened to some extent. Under the condition of constant load, we believe that the shortening of femoral offset (the vertical distance between the center of rotation of the femoral head and the longitudinal axis of the femoral shaft) is a major factor for the variation of the bending moment effect as the arm of force changes accordingly.

According to Wolff's law, the adaptive bone remodeling of the femur is unavoidable in accordance with the new biomechanical environment, thus leading to bone loss, cortical bone thinning, cortical bone area reduction, bone mineral density (BMD) decline, and prosthesis loosening due to interfacial gaps [14, 38–41]. As the actual stress of the bone is greater than the optimal stress, the bone formation is dominant. Otherwise, the bone absorption is dominant because of less stress stimulation. Bayraktar et al. [26] used several theoretical models to explain the effects of external load on the BMD and its trabecular structure. The bone remodeling around the prosthesis was predicted, and the results of stress shielding combined with the design of the prosthesis were evaluated.

Previous study has shown that the largest area of the cortical bone loss for the cementless prosthesis is located in the proximal and middle regions with a rate of 40% [42]. According to the four quadrants, the area is located in the proximal medial region with a loss of 55%. It is interesting to observe that the results of our study may be different from the above-mentioned conclusions. This difference is probably due to the design principles of the prosthesis. It is clearly stated in the specification that this stem is designed with deep grooves to increase the modulus of elasticity and to reduce the stress shielding effect or excessive stiffening of the proximal femur caused by the metallic implant. Deep grooves reducing the cross section of the stem provide a “constructive elasticity” together with the favorable modulus of elasticity of the titanium alloy, thus reducing the stress shielding. Moreover, an anchoring screw through a bore hole in the lateral fin can be screwed into the greater trochanter to reduce the compressive load onto the calcar during the initial postoperative stage. Given this perspective, it is reasonable to explain the fact that the stress of the medial region is dispersed. More importantly, our results also confirmed the study of Wu et al. about the BMD changes around the prosthesis [17]. They found that there was a statistical difference in BMD changes in Gruen zones 4 and 5 [17]. Silva et al. proposed that severe bone loss was likely to occur as the stress shielding rate was more than 30% and our data of multiregion showed a higher rate [43]. Therefore, for patients undergoing postoperative review, special attention should be paid to observe the BMD around the prosthesis and various interfaces to find possible early loosening.

The results of this study showed that the stress shielding area of the Ribbed prosthesis was mainly located in the posterior region. On account of the porous coating limited to the proximal section, the design solved well the problem of proximal stress shielding caused by the stress concentration in the middle and distal regions, but our data indicated that the prosthesis still had some inherent defects. Katoozian and Davy focused on the idealized prosthesis similar to physiological condition from a purely mechanical point of view [44]. As a result, the prosthesis morphology was not regular and could not be further clinically applied due to the individual differences and the complexity of the internal load conditions. Adaptive bone remodeling secondary to stress shielding is also considered to be associated with ipsilateral femoral fractures, limb pain, and poor function [45]. The maximum stress value of the femur appeared at the end of the prosthesis after implantation, and the thigh pain might be associated with the uneven pressure of periosteal in that region. We believe that the implantation of a neutral prosthesis can avoid the stress concentration to a great extent at the end of the prosthesis, and the varus or valgus insert should not be allowed.

The limitations of our study are as follows. (i) The stress distribution in each region was not analyzed in combination with the corresponding BMD, so some convincing solutions to the design defects of prosthesis could not be proposed. (ii) More samples are needed to determine the biomechanical variation in overall femoral stress and periprosthetic femoral stress distribution after implantation. (iii) Since we did not have an optimal technique in clearing off the metal artifacts to avoid impact on results, we selected the contralateral side. With the rapid development of related software, this problem may be solved to make the FEA much closer to the real condition.

## 5. Conclusion

In conclusion, the stress changes in magnitude and distribution of the periprosthetic bone tissue may be the main causes of bone loss and aseptic loosening. Ideal prosthesis should achieve good stability after implantation without severe stress shielding. The prosthesis design still needs to be improved from the surface of the porous coating, the geometry of the prosthesis, to the elastic modulus of the material. Our study may help to optimize the prosthetic design to reduce stress shielding effect and improve clinical outcomes.

## Figures and Tables

**Table 1:** Material properties applied in the FEA model.

Material	Elastic modulus (GPa)	Poisson's ratio	Mass densities (g/cm^3^)
Cortical bone	*E*_*x*_, *E*_*y*_ = 7.0; *E*_*z*_ = 11.5, *G*_*xy*_ = 2.6; *G*_*yz*_, *G*_*zx*_ = 3.5	0.4	1.99
Cancellous bone	0.4	0.3	0.05
Titanium alloy	109	0.28	4.51
CoCrMO alloy	210	0.3	8.62

## Data Availability

The data used to support the findings of this study are available from the corresponding author upon request.
